# ATP Sensitive Potassium Channels in the Skeletal Muscle Function: Involvement of the *KCNJ11*(Kir6.2) Gene in the Determination of Mechanical Warner Bratzer Shear Force

**DOI:** 10.3389/fphys.2016.00167

**Published:** 2016-05-10

**Authors:** Domenico Tricarico, Maria Selvaggi, Giuseppe Passantino, Pasquale De Palo, Cataldo Dario, Pasquale Centoducati, Alessandra Tateo, Angela Curci, Fatima Maqoud, Antonietta Mele, Giulia M. Camerino, Antonella Liantonio, Paola Imbrici, Nicola Zizzo

**Affiliations:** ^1^Department of Pharmacy-Drug Science, University of Bari Aldo MoroBari, Italy; ^2^Section of Veterinary Science and Animal Production, Department of Emergency and Organ Transplantation (DETO), University of Bari Aldo MoroValenzano, Italy; ^3^Department of Veterinary Medicine, University of Bari Aldo MoroBari, Italy; ^4^Faculty of Science, Chouaib Doukkali UniversityEl Jadida, Morocco

**Keywords:** meet tenderness, ATP sensitive K+ channels, Warner-Bratzler shear force, skeletal muscle, gene polymorphisms

## Abstract

The ATP-sensitive K^+^-channels (KATP) are distributed in the tissues coupling metabolism with K^+^ ions efflux. KATP subunits are encoded by *KCNJ8* (Kir6.1), *KCNJ11* (Kir6.2), *ABCC8* (SUR1), and *ABCC9* (SUR2) genes, alternative RNA splicing give rise to SUR variants that confer distinct physiological properties on the channel. An high expression/activity of the sarco-KATP channel is observed in various rat fast-twitch muscles, characterized by elevated muscle strength, while a low expression/activity is observed in the slow-twitch muscles characterized by reduced strength and frailty. Down-regulation of the KATP subunits of fast-twitch fibers is found in conditions characterized by weakness and frailty. *KCNJ11* gene knockout mice have reduced glycogen, lean phenotype, lower body fat, and weakness. KATP channel is also a sensor of muscle atrophy. The *KCNJ11* gene is located on BTA15, close to a QTL for meat tenderness, it has also a role in glycogen storage, a key mechanism of the postmortem transformation of muscle into meat. The role of *KCNJ11* gene in muscle function may underlie an effect of *KCNJ11* genotypes on meat tenderness, as recently reported. The fiber phenotype and genotype are important in livestock production science. Quantitative traits including meat production and quality are influenced both by environment and genes. Molecular markers can play an important role in the genetic improvement of animals through breeding strategies. Many factors influence the muscle Warner-Bratzler shear force including breed, age, feeding, the biochemical, and functional parameters. The role of *KCNJ11*gene and related genes on muscle tenderness will be discussed in the present review.

## Introduction

The ATP-sensitive K^+^-channels (KATP) are widely distributed in the tissues including neurons, vascular, pancreatic beta cells, cardiac, and skeletal muscles (Amoroso et al., 1990; Zhang and Bolton, 1996; Liss and Roeper, 2001; Cole and Clément-Chomienne, 2003; Flagg et al., 2010; Olson and Terzic, 2010). The multi-level regulation by membrane phospholipids (PIP2), fatty acids (LC-Acyl-CoA), protein kinases (PKA, PKC), creatin kinase shuttle, and glycolytic enzymes, pH, hypoxia, and intracellular nucleotides ensures complexity of metabolic sensing by KATP channels (Selivanov et al., 2004; Flagg et al., 2010; Mele et al., 2012; Mohammed Abdul et al., 2015). The main regulator of the KATP channel is the ATP/ADP ratio, an elevated ATP/ADP ratio leads to channel closure while the reduction of the ATP/ADP ratio in the presence of Mg^2+^ ions determine the channel opening thereby sensing nucleotides changes (Flagg et al., 2010).

### Structure, distribution, and regulation of KATP channels

The KATP channels are hetero-octameric complexes of pore-forming inwardly rectifier K^+^ (Kir6) channel subunits associated with regulatory sulphonylureas receptor (SUR) subunits, members of the ATP binding cassette (ABC) family of membrane proteins. Two Kir6-encoding genes, *KCNJ8* (Kir6.1) and *KCNJ11*(Kir6.2), and two SUR genes, *ABCC8* (SUR1) and *ABCC9* (SUR2), encode mammalian KATP subunits, but alternative RNA splicing can give rise to multiple SUR protein variants (e.g., SUR2A and SUR2B) that confer distinct physiological and pharmacological properties on the channel complex (Inagaki et al., 1995, 1996; Chutkow et al., 1996; Babenko et al., 2000; Tricarico et al., 2006; Wheeler et al., 2008). The nucleotide inhibitory and stimulatory sites are located on the Kir6.2/Kir6.1 and on SURs subunits of the channel complex, respectively (Babenko et al., 2000; Flagg et al., 2010).

The SUR subunits carry the binding sites for the KATP channel blockers used as insulin releasing agents, and for the KATP channel openers used as cardioprotective and vasodilating drugs (Babenko et al., 2000; Tricarico et al., 2008a, 2012). These drugs are also effective on the skeletal muscle KATP channels (Table 1).

**Table 1:** **Molecular composition and functions of KATP channel subunits in skeletal muscles**.

**Fiber types and properties**	**Molecular composition of sarco KATP channels**	**Functions**	**Pharmacology**
**Fast-twitch muscles**		Elevated expression/activity.	Responsive to openers and blockers.
type IIB glycolitic fibers, show low mitochondria density, are resistant to mechanical stress, fatigue sensitive, are involved in the short term intense explosive exercise (min).	Kir6.2/SUR2A> Kir6.2/SUR2B>> Kir6.2/SUR1	Partial cytoprotection against disuse related atrophy and staurosporine induced atrophy. Upregulation of SUR2A in response to hypoxia. Downregulation of Kir6.2/SUR2A in response to hypokalemia with weakness. Reduced activity with aging.	Rank order of efficacy of the KATP openers: benzoxazine analogs>>cromakalim = minoxidil = pinacidil> diazoxide Rank order of efficacy of the KATP blockers: glimepiride≥glibenclamide> repaglinide>nateglinide> tolbutamide
Type IIA glycolitic-oxidative fibers, show low sensitivity to fatigue, are involved in the moderate intensity exercise, and resistance to elevated mechanical stress. Type IIX fibers with intermediate properties between type IIB and IIA fibers.	Kir6.2/SUR2A> Kir6.2/SUR1>> Kir6.2/SUR2B	Full cytoprotection against muscle disuse and staurosporine induced atrophy. Downregulation of Kir6.2/SUR2A in response to hypokalemia with weakness. Reduced activity with aging.	Rank order of efficacy of the KATP openers: benzoxazine analogs >>cromakalim = minoxidil = pinacidil = diazoxide Rank order of efficacy of the KATP blockers: glibenclamide>repaglinide> nateglinide>glimepiride> tolbutamide
**Slow twitch muscles**		Reduced expression/activity.	Less responsive to openers and blockers.
Type I oxidative fibers show high mitochondria density, elevated frailty, fatigue resistance, and recruited following low intensity and long lasting exercise (hours).	Kir6.2/SUR2A> Kir6.2/SUR2B≥ Kir6.2/SUR1	Enhanced susceptibility to atrophic stressors. Downregulation of Kir6.2/SUR2B, SUR1 in response to muscle disuse and staurosporine induced atrophy. In the short term upregulation of Kir6.2/SUR2B/SUR1 with cytoprotection against staurosporine insults.	Rank order of efficacy of the KATP openers: cromakalim > diazoxide Rank order of efficacy of the blockers: repaglinide>glibenclamide≥ nateglinide>tolbutamide> glimepiride

*The gene expression of the KATP channel subunits and the sarcolemma KATP channels (sarco-KATP) activity have been evaluated, respectively, by RT-PCR experiments in muscles and in excised macropatch experiments performed on enzymatically isolated native rat fibers. The drug effects on the channel currents were evaluated in symmetrical concentrations of K^+^ ions on both side of the membrane patches, at −60 mV(Vm), in the absence of internal ATP and channel blockers or in the presence of internal ATP (100 10^−6^ M) and channel openers*.

As in cardiac muscle, skeletal muscle KATP channels (sarco-KATP) remain closed at rest and do not contribute to electrical activity unless the muscle is stressed. Channel regulation by intracellular nucleotide, metabolic enzymes, and ATP-ase pumps are similar to that in cardiac muscle, but the intracellular acidification is a potent activator of the skeletal muscle subtype (Tricarico et al., 1997a, 2003, 2012).

The properties of the sarco-KATP channels are age dependent in rat fibers. The activity recorded in excised patches from fast-twitch fibers is low at 5–6 days of postnatal life, increases to a plateau at 12–13 days, then declines toward adult values after 37 days. Two distinct types of the KATP channel complex can be distinguished. The early developmental period (5–6 days) is dominated by a KATP channel having a conductance of 66 pS, a high open probability of 0.602 which is determined by a reduced mean close time as compared to that recorded in the adult fibers, and an IC50 for ATP and glybenclamide of 123.1 and 3.97 μM, respectively. The later developmental period (from 56 days) is dominated by a KATP channel having a 71 pS conductance, but a low open probability of 0.222. This adult channel is also 3.2 and 73.5 times more sensitive to ATP and glybenclamide than the juvenile channel, respectively (Tricarico et al., 1997b).

The molecular composition of the sarco-KATP channels has been clarified in adult rat muscle fibers. Hybrid KATP channel complexes composed of Kir6.2, SUR2A, SUR1, and SUR2B subunits contribute to functional channels in different muscle phenotypes (Tricarico et al., 2006). A high expression/activity of the Kir6.2-SUR2A and Kir6.2-SUR1 channel subunits is observed in type IIA fast-twitch muscles, characterized by elevated strength. A low expression/activity of the sarco-KATP channel is observed in the slow-twitch muscle of the rat characterized by reduced strength and frailty being more susceptible to mechanical and chemical insults, and the Kir6.2-SUR2B subunits contribute to the functional channel in this muscle phenotype (Table 1). The sarco-KATP channel activity declines with aging in fast-twitch rat fibers showing surface channel subtypes characterized by low open probability and current density (Tricarico and Camerino, 1994).

The age-dependent changes of the KATP channels subtypes may reflect the different metabolic needs of the muscles during development and aging.

### Role of KATP channels in skeletal muscle homeostasis and fatigue

The role of the sarco-KATP channels in the muscle fatigue has been extensively investigated. Muscle fatigue is the decline in force production during prolonged and repetitive stimulation and many biochemical mechanisms have been proposed to contribute to this process. One possible mechanism is that activation of KATP channels, in response to reduction of ATP/ADP ratio, might underlie a decrease in action potential duration and hence twitch force. The activation of sarco-KATP channels after fatigue has developed, helps to preserve a polarized membrane potential and rise of tension that is observed in Kir6.2^−∕−^ muscles that are exposed to fatiguing stimuli (Gramolini and Renaud, 1997; Gong et al., 2000, 2003; Cifelli et al., 2007, 2008). KATP channels play a role in Ca^2+^ handling and maintaining fiber integrity during exercise. Abolishing KATP channel activity in fast-twitch muscle fibers leads to a decrease in peak Ca^2+^ and tetanic force, increases in resting unstimulated Ca^2+^ ions with faster fatigue rate (Cifelli et al., 2007). The observation that there is extensive fiber damage in Kir6.2^−∕−^ subjected to training protocols corroborates the conclusion that KATP channel activation is a physiologically relevant myoprotective mechanism *in vivo* (Kane et al., 2004; Thabet et al., 2005). These findings accounts for the observation that the rate and extent of post-fatigue recovery are decreased in Kir6.2^−∕−^animals. A similar phenotype has been found in SUR2^−∕−^mice, that show impaired exercise performance and extensive fiber damage following exercise (Stoller et al., 2009).

**Figure 1:**
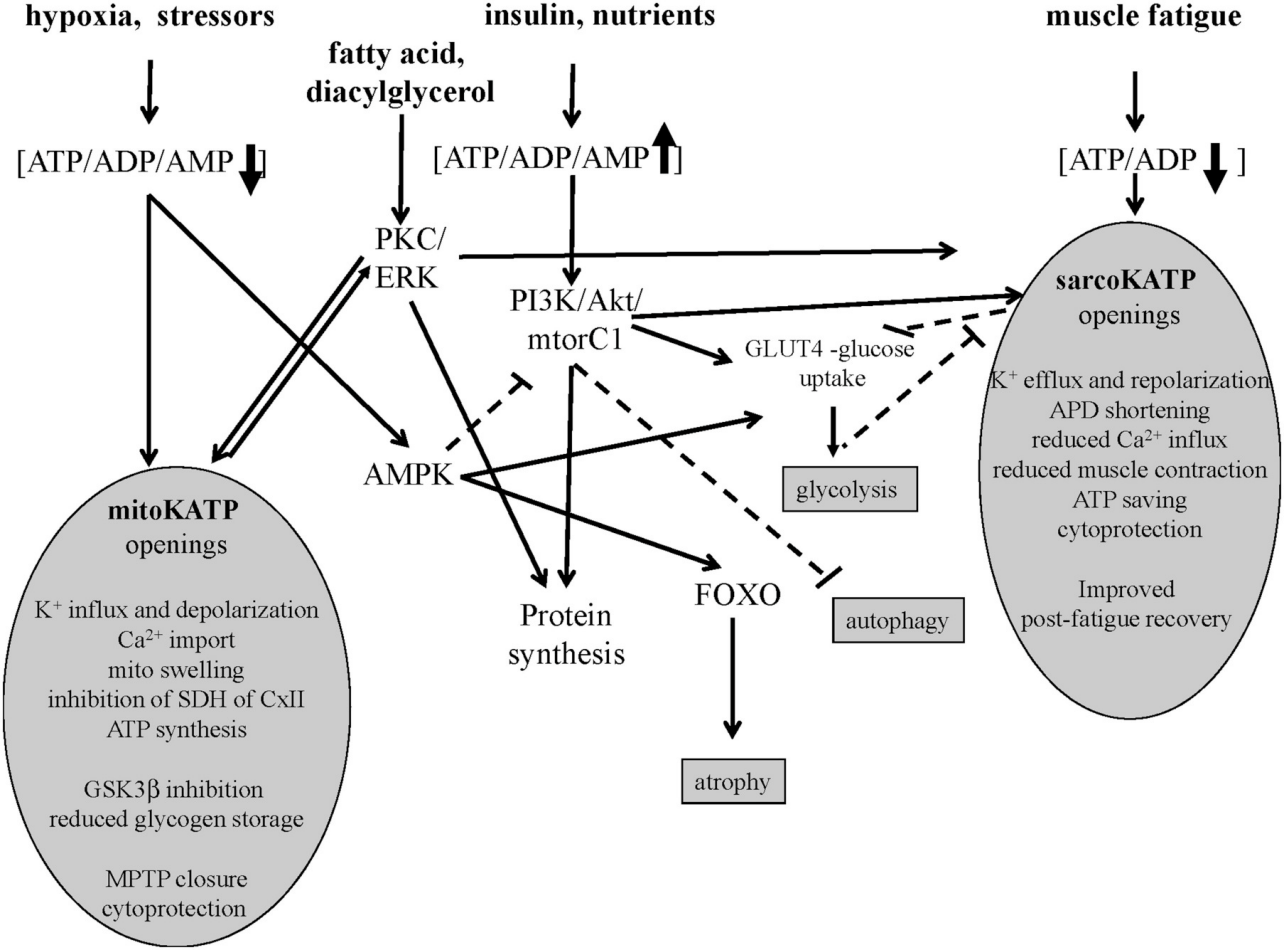
**Signaling pathways of sarco-KATP and mito-KATP channels in skeletal muscle**. Hypoxia-induced lowering of ATP/ADP/AMP ratio activates mito-KATP channel with mito-swelling, inhibition of succinic dehydrogenase(SDH) of complex II, ATP synthesis, inhibition of glycogen-synthase-kinase-3beta(GSK3β), closure of the mitochondrial permeability transition pore(MPTP). The high ATP/ADP/AMP ratio activates the phosphatidylinositol-3 kinase/protein kinase B /mammalian target of rapamycin complex1(mtorC1) (PI3K/Akt/mtorC1) pathway. The AMP-activated protein-kinase(AMPK) inhibits mtorC1 and activates forkhead box protein O(FOXO). The lowering of ATP/ADP ratio following fatigue activates the sarco-KATP channels with improved post-fatigue recovery. In slow twitch fibers, protein kinase C(PKC) and extracellular signal-regulated kinases(ERK) may activates sarco-KATP and mito-KATP channels with cytoprotection. Continuous line indicates activation, dashed line inhibition.

The sarco-KATP channels regulate glucose homeostasis. The *in vitro* pharmacological blockade of sarco-KATP channels in cell line as well as the down-regulation of the subunits increases basal or insulin-dependent glucose uptake as observed in KATP deficient Kir6.2^−∕−^ and SUR2^−∕−^ animals (Tsiani et al., 1995; Chutkow et al., 2001; Wasada et al., 2001). In these mice there is enhanced glucose uptake, consistent with an inhibitory effect of sarco-KATP activity on glucose uptake (Wasada, 2002). *KCNJ11* gene knockout mice (Kir6.2^−∕−^) also have reduced glycogen storage, lean phenotype, lower body fat, and severe muscle weakness (Alekseev et al., 2010; Figure 1). Down-regulation of Kir6.2/SUR2A subunits in skeletal muscle is associated with abnormal insulin response with severe hypokalemia and hypoglycaemia in rats (Tricarico et al., 2003, 2008b). It may be possible that the abnormally enhanced KATP channel activity as occurring in neonatal diabetes will affect also glucose uptake into skeletal muscle, thereby exacerbating the hyperglycaemia (Ellard et al., 2007; McTaggart et al., 2010). Similarly, in obesity an accumulation of fatty acyl-CoA intermediates, by activating KATP channels will exacerbate insulin resistance (Wasada et al., 2001; Wasada, 2002).

The phenotype-dependent KATP activity therefore leads to a better use of glucose among muscles in proportion to their metabolic needs. The enhanced expression/activity of the sarco-KATP channels reduces the glucose uptake in low energetic demand fast-twitch muscles, while making glucose available to slow-twitch muscles which are characterized by a high glucose demand during contraction and reduced expression/activity of the sarco-KATP channels (Bonen et al., 1981; Megeney et al., 1993; Tricarico et al., 2006). This mechanism may contribute to the action of the KATP channels in regulating the rate and extent of post-fatigue recovery.

The observed differences in KATP channel properties among muscles in terms of expression/activity and composition of channel subunits can be related to their specific functions in rat (Tricarico et al., 2006). The KATP channels of *flexor digitorum brevis (FDB)* are indeed composed of SUR1 and SUR2 subunits, whereas KATP channels of *tibialis anterioris (TA), extensor digitorum longus (EDL)*, and *soleus (SOL)* muscles are composed of SUR2 subunits which are more responsive to metabolic stresses compared with channel complexes of SUR1. The *TA, EDL, and SOL* muscles are exposed more often to hypoxia and fatigue than *FDB* muscle, which shows a different morphology and function (Table 1).

### KATP channel subunits regulate apoptosis and cell viability

The *in vivo* down-regulation of KATP channel subunits or the *in vitro* long term exposure of the channels to channel blockers (>24 h) are coupled to apoptosis and atrophic signaling in isolated fibers (Tricarico et al., 2010; Cetrone et al., 2014; Mele et al., 2014a,b). The atrophic effects of the channel blockers and of the apoptotic agent staurosporine are muscle type dependent and are related with the sarco-KATP channel density. For instance, the KATP channel blockers induces atrophy after 24 h of incubation time of fast-twitch fibers that are characterized by elevated sarco-KATP channels expression/activity, while the apoptotic agent staurosporine induce atrophy within 6 h of incubation time affecting slow-twitch fibers that are characterized by low sarco-KATP channel expression/activity. These findings corroborate the idea that the high/expression activity of the sarco-KATP channel subunits is a protective factor against insults (Cetrone et al., 2014; Mele et al., 2014a).

Emerging evidences suggest that in skeletal and cardiac muscles the sarco-KATP and mitochondria KATP (mito-KATP) channels are coupled to the insulin/IGF1-PI3K-Akt-mtor signaling and/or PKC/ERK pathway (Figure 1). The SUR2A gene is upregulated by Akt following hypoxia and exerts cytoprotective action saving intracellular ATP/ADP/AMP ratio which is critical in regulating the Akt–mtor pathway (Vadlakonda et al., 2013; Mohammed Abdul et al., 2015). The Akt–mtorC1 pathway determines protein synthesis also inhibiting FOXO atrophic signaling in fast-twitch skeletal muscle (Bonaldo and Sandri, 2013).

The sarco-KATP and mito-KATP channels are modulated by PKC phosphorylation in cardiomyocite and in cell line expressing the recombinant channels (Light et al., 2000). PKC is coupled to ERK in skeletal muscle (Ronda et al., 2010). The fact that the unselective PKC inhibitor staurosporine induces atrophy of slow fibers which is prevented by diazoxide, suggests that PKC/ERK plays a role in this muscle phenotype in regulating protein synthesis (Mele et al., 2014a).

Diazoxide activates mito-KATP, potentiates PKCε, and PI3K/Akt/mtorC1 pathways (Kim et al., 2006; Kwon et al., 2006; Chen et al., 2016). While, the mito-KATP channel blocker 5-hydroxydecanoate (5HD) and the sulphonylureas show opposite actions (Khanfar et al., 2013; Mele et al., 2014b).

The opening of mito-KATP channel following low ATP levels induces mitochondrial swelling of the inner membrane regulating ATP synthesis (Wojtovich et al., 2013). The mito-KATP channel was initially identified into the SUR1/Kir6.1 complex that recapitulated mito-KATP channel activity, including diazoxide activation and 5-hydroxydecanoate inhibition (Ardehali et al., 2004). Recently, a role for ROMK2 (Kir1) subunit in generating the mito-KATP channel has been proposed (Foster et al., 2012). Openings of the mito-KATP channel blocks the mito-permeability transition pore reducing cytochrome C release in different cells. The mito-KATP channel is also coupled to the glycogen-synthase-kinase 3beta (GSK-3β) and connexin 43(Cx43) so that the GSK-3β downregulation transfers cytoprotective signaling through mitochondrial Cx43 onto mito-KATP channels openings (Rottlaender et al., 2012; Figure 1).

### KATP channelophaties

The KATP channels are involved in rare genetic diseases associated with insulin/glucose dis-metabolism, cardiomyopathy, weakness, and dysmorphisms.

Mutations in the *KCNJ11* and *ABCC8* genes are now well-understood to underlie neonatal diabetes mellitus and congenital hyperinsulinism (Ellard et al., 2007; Flanagan et al., 2007; Arnoux et al., 2010; McTaggart et al., 2010). Activating mutations in the *KCNJ11* gene encoding for the Kir6.2 subunit is associated with severe neuro-muscular weakness in permanent neonatal diabetes (Gloyn et al., 2004).

The SUR1 and SUR2A/B subunits are involved in neuroprotection following ischemia, and in neurodegenerative diseases such as Parkinson or Alzheimer's diseases and aging (Liss and Roeper, 2001; Zeng et al., 2007; Nelson et al., 2015). Gain of function and loss of function mutations in the *KCNJ8* gene is associated with the J-wave phenomenon and early repolarization of the hearth, and with the sudden infant death syndrome, respectively (Kane et al., 2005; Nichols et al., 2013). Loss-of-function mutations of the *ABCC9* gene were found in patients affected by long-standing atrial fibrillation originating in the vein of Marshall and in patients with dilated cardiomyopathy (Kane et al., 2005; Nichols et al., 2013).

Down regulation of the KATP channel subunits of fast-twitching fibers is associated with hypokalemic periodic paralysis. This disorder is characterized by attacks of weakness induced by insulin-glucose infusion and lowering of serum K^+^ ions (Tricarico et al., 1998, 1999, 2008b).

Gain of function mutations in the *ABCC9* gene encoding for the cardiac, skeletal and smooth muscles SUR2A/B subunits of the KATP channel is responsible for the Cantu' syndrome, a distinctive multi-organ disease characterized by hypertrichosis, osteochondrodysplasia, cardiomegaly, and musculo-skeletal abnormalities (Harakalova et al., 2012).

### Involvement of the *KCNJ11*(Kir6.2) gene in the determination of the meat tenderness

Several polymorphisms were detected at *KCNJ11 locus* in a sample of Nellore cattle (Tizioto et al., 2013). Among them, only two SNPs were used to investigate a possible association with meat tenderness of meat by using the Warner-Bratzler shear force (WBSF). The first polymorphism, c.1526C>T(NCBL_ss#537718973) is a synonymous mutation located in the coding region; the second one, c.2342T>C(NCBL_ss#537718995) is located in the 3′UTR region. The SNP c.2342T>C showed an additive effect on WBSF measured 24 h after slaughter and after 7 days of cold-chamber aging, with the T allele being associate with reduced WBSF. No effect of the haplotype was observed (Tizioto et al., 2013). The *KCNJ11* gene is located on bovine chromosome 15, near a quantitative trait locus (QTL) for meat tenderness thereby indicating the involvement of this gene in regulating this muscle parameter in bovine species (Rexroad et al., 2001; Tizioto et al., 2013).

These findings suggest that the expression/activity of the KATP channel subunits may have relevance in determining the WBSF in muscles also under different nutritional status (De Palo et al., 2012). Meat tenderness is affected by breed, gender, and nutritional status. Biochemical and functional analysis showed that lower shear force values were associated with more tender meat. Muscles in the highest tenderness cluster had the lowest total and insoluble collagen contents, the highest mitochondrial enzyme activity (isocitrate dehydrogenase), the highest proportion of slow oxidative fibers, the lowest proportion of fast-glycolytic fibers, the lowest average muscle fiber cross-sectional area and showing intramuscular fat (Chriki et al., 2012).

Gene and proteomic analysis identified several gene pathways associated with tenderness, among these the heat shock proteins, the calpain/calpastatin and apoptotic genes, the energy and metabolic genes, and fatty acid related genes pathways have attracted the attention of several investigators (Hocquette et al., 2012; Picard et al., 2014). However, it is not always possible to extrapolate the relevance of these markers to all bovine population.

Moreover, a significant relationship between the *KCNJ11* gene expression level and the WBSF after 7 days of cold-chamber aging was found, without a significant influence of the investigated SNPs on gene expression levels (Tizioto et al., 2013). Although, the *KCNJ11 gene* encodes for a K^+^ channels Tizioto and coworkers reported no significant association between the two considered SNPs and the K^+^ content in the meat obtained by Nellore cattle in Brazil. However, as these authors state, they did not distinguish between intracellular and extracellular K^+^ content, being impossible to detect the regulation of K^+^ flow (Tizioto et al., 2014). So, to further investigate all these concerns also in other breeds, genetically distant from Nellore, could be an interesting perspective.

The role of K^+^ ions content on meet tenderness is controversial. It seems that higher levels of K^+^ ions are related to meat tenderness, instrumentally evaluated through trained panel test (Mateescu et al., 2013). The addition of K-lactate in fresh bovine chuck muscles confers more tenderness to meat (Walsh et al., 2010). Furthermore, the substitution of Na^+^ with K^+^ ions brought to a higher tenderness of the product evaluating sensorial profile modifications through panel test (Greiff et al., 2015). Although, mechanical and instrumental meat tenderness determination is not strictly correlated to sensorial evaluation of the same qualitative pattern, different authors recently found a positive correlation between meat tenderness and K^+^ ions concentration. Similar results obtained with different tenderness evaluation techniques represent a strong confirmation of results. Actually scientific community is discussing on the best way for evaluating this important qualitative pattern of meat sensorial profile, comparing and correlating results obtained by human based analysis (consumer and panel tests), mechanical analysis (WBSF and Meullenet–Owens razor shear) and spectroscopy (visible and near-infrared; Yancey et al., 2010; Emerson et al., 2013). Besides, some found in Angus breed a heritability rate of K^+^ ions concentration indicating also an important role of genetics in this aspect (Mateescu et al., 2013). Knock outs mice of K^+^ channel gene subunits other than KATP subunits show a similar phenotype (muscle weakness, small size) supporting the role of K^+^ ions as a factor involved in the muscle tenderness. Low intracellular K^+^ content may activates K-dependent proteolysis effectors such as caspases with a potential role in the muscle tenderness (Nolin et al., 2016). Moreover, muscle-specific signaling pathways coupled to the splicing forms of the KATP channels may play a role (Figure 1).

## Conclusions

Several critical questions remain open for instance the role and distribution of *KCNJ11* gene polymorphisms and related genes in the Italian cattle breeds which has never been investigated. The effects of the c.2342T>C and c.1526C>T SNPs at *KCNJ11 locus* on the expression/activity of the sarco-KATP channel subunits in *ex vivo* experiments as well as the cellular phenotype changes associated with the *KCNJ11* gene down-regulation in the muscles and in different native cell types of cattle are not known.

The correlation of the fiber phenotypes using biochemical and histological analysis of the muscle in terms of fiber composition, fat and collagen contents, qualitative and chromatic aspects with gene expression, and polymorphisms is another point of interest (Martin et al., 1985; Schiaffino et al., 1989; Picard et al., 1998; Duris et al., 2002; Tateo et al., 2007).

Genetic prediction of beef tenderness in bovine breeds represents an important topic useful to widen knowledge on the complex phenomena related to mechanical and sensorial properties of fresh and cured meat, but it could be a fundamental tool necessary for genetic improvement of meat producing animals, and of beef at first.

## Author contributions

DT, wrote the manuscript. AL, Revision of the manuscript. PI, Editing. AC, AM Cellular experiments. NZ, GP, Pathology. MS, CD, Genetists. PD, MS, Wrote the manuscript. AT, PC, PD, Quality control. GC, Molecular biology.

### Conflict of interest statement

The authors declare that the research was conducted in the absence of any commercial or financial relationships that could be construed as a potential conflict of interest.

## References

[B1] Alekseev A. E. Reyes S. Yamada S. Hodgson-Zigman D. M. Zhu S. Z. Sirerra A.. (2010). Sarcolemmal ATP-sensitive K^+^ channels control energy expenditure determining body weight. Cell Metab. 11, 50–69. 10.1016/j.cmet.2009.11.009 20074528 PMC2849280

[B2] Amoroso S. Schmid-Antomarchi H. Fosset M. Lazdunski M. (1990). Glucose, sulfonylureas, and neurotransmitter release: role of ATP-sensitive K^+^ channels. Science 247, 852–854. 10.1126/science.2305257 2305257

[B3] Ardehali H. Chen Z. Ko Y. Mejía-Alvarez R. Marbán E. (2004). Multiprotein complex containing succinate dehydrogenase confers mitochondrial ATP-sensitive K+ channel activity. Proc. Nat. Acad. Sci. U.S.A. 101, 11880–11885. 10.1073/pnas.0401703101 15284438 PMC511068

[B4] Arnoux J. B. de Lonlay P. Ribeiro M. J. Hussain K. Blankenstein O. Mohnike K.. (2010). Congenital hyperinsulinism. Early Hum. Dev. 86, 287–294. 10.1016/j.earlhumdev.2010.05.003 20550977

[B5] Babenko A. P. Gonzalez G. Bryan J. (2000). Pharmaco-topology of sulfonylurea receptors. Separate domains of the regulatory subunits of K(ATP) channel isoforms are required for selective interaction with K(+) channel openers. J. Biol. Chem. 275, 717–720. 10.1074/jbc.275.2.717 10625598

[B6] Bonaldo P. Sandri M. (2013). Cellular and molecular mechanisms of muscle atrophy. Dis. Model. Mech. 6, 25–39. 10.1242/dmm.010389 23268536 PMC3529336

[B7] Bonen A. Tan M. H. Watson-Wright W. M. (1981). Insulin binding and glucose uptake differences in rodent skeletal muscles. Diabetes 30, 702–704. 10.2337/diab.30.8.702 7018974

[B8] Cetrone M. Mele A. Tricarico D. (2014). Effects of the antidiabetic drugs on the age-related atrophy and sarcopenia associated with diabetes type II. Curr. Diabetes Rev. 10, 231–237. 10.2174/1573399810666140918121022 25245021

[B9] Chen W. Liu Y. Xue G. Zhang L. Zhang L. Shao S. (2016). Diazoxide protects L6 skeletal myoblasts from H2O2-induced apoptosis via the phosphatidylinositol-3 kinase/Akt pathway. Inflamm. Res. 65, 53–60. 10.1007/s00011-015-0890-1 26525360

[B10] Chriki S. Gardner G. E. Jurie C. Picard B. Micol D. Brun J.-P.. (2012). Cluster analysis application identifies muscle characteristics of importance for beef tenderness. BMC Biochem. 13:29. 10.1186/1471-2091-13-29 23259756 PMC3544649

[B11] Chutkow W. A. Samuel V. Hansen P. A. Pu J. Valdivia C. R. Makielski J. C.. (2001). Disruption ofSur2-containing K(ATP) channels enhances insulin-stimulated glucose uptake in skeletal muscle. Proc. Natl. Acad. Sci. U.S.A. 98, 11760–11764. 10.1073/pnas.201390398 11562480 PMC58803

[B12] Chutkow W. A. Simon M. C. Le Beau M. M. Burant C. F. (1996). Cloning, tissue expression, and chromosomal localization of SUR2, the putative drug-binding subunit of cardiac, skeletal muscle, and vascular KATP channels. Diabetes 45, 1439–1445. 10.2337/diab.45.10.1439 8826984

[B13] Cifelli C. Boudreault L. Gong B. Bercier J. P. Renaud J. M. (2008). Contractile dysfunctions in ATP dependent K^+^ channel-deficient mouse muscle during fatigue involve excessive depolarization and Ca^2+^ influx through L-type Ca^2+^ channels. Exp. Physiol. 93, 1126–1138. 10.1113/expphysiol.2008.042572 18586858

[B14] Cifelli C. Bourassa F. Gariepy L. Banas K. Benkhalti M. Renaud J. M. (2007). KATP channel deficiency in mouse flexor digitorum brevis causes fibre damage and impairs Ca^2+^ release and force development during fatigue *in vitro*. J. Physiol. 582, 843–857. 10.1113/jphysiol.2007.130955 17510189 PMC2075337

[B15] Cole W. C. Clément-Chomienne O. (2003). ATP-sensitive K^+^ channels of vascular smooth muscle cells. J. Cardiovasc. Electrophysiol. 14, 94–103. 10.1046/j.1540-8167.2003.02376.x 12625619

[B16] De Palo P. Maggiolino A. Centoducati P. Tateo A. (2012). Colour changes in meat of foals as affected by slaughtering age and post-thawing time. Asian-Australas. J. Anim. Sci. 25, 1775–1779. 10.5713/ajas.2012.12361 25049544 PMC4094156

[B17] Duris M.-P. Picard B. Geay Y. (2002). Specificity of different antimyosin heavy chain antibodies in bovine muscle. Meat Sci. 55, 67–78. 10.1016/S0309-1740(99)00127-8 22060906

[B18] Ellard S. Flanagan S. E. Girard C. A. Patch A. M. Harries L. W. Parrish A.. (2007). Permanent neonatal diabetes caused by dominant, recessive, or compound heterozygous SUR1 mutations with opposite functional effects. Am. J. Hum. Genet. 81, 375–382. 10.1086/519174 17668386 PMC1950816

[B19] Emerson M. R. Woerner D. R. Belk K. E. Tatum J. D. (2013). Effectiveness of USDA instrument-based marbling measurements for categorizing beef carcasses according to differences in longissimus muscle sensory attributes. J. Anim. Sci. 91, 1024–1034. 10.2527/jas.2012-5514 23148250

[B20] Flagg T. P. Enkvetchakul D. Koster J. C. Nichols C. G. (2010). Muscle KATP channels: recent insights to energy sensing and myoprotection. Physiol. Rev. 90, 799–829. 10.1152/physrev.00027.2009 20664073 PMC3125986

[B21] Flanagan S. E. Patch A. M. Mackay D. J. Edghill E. L. Gloyn A. L. Robinson D.. (2007). Mutations in ATP-sensitive K^+^ channel genes cause transient neonatal diabetes and permanent diabetes in childhood or adulthood. Diabetes 56, 1930–1937. 10.2337/db07-0043 17446535 PMC7611811

[B22] Foster D. B. Ho A. S. Rucker J. J. Garlid A. O. Chen L. Sidor A.. (2012). Mitochondrial ROMK channel is a molecular component of mitoKATP. Circ. Res. 111, 446–454. 10.1161/CIRCRESAHA.112.266445 22811560 PMC3560389

[B23] Gloyn A. L. Pearson E. R. Antcliff J. F. Proks P. Bruining G. J. Slingerland A. S.. (2004). Activating mutations in the gene encoding the ATP-sensitive potassium-channel subunit Kir6.2 and permanent neonatal diabetes. N. Engl. J. Med. 350, 1838–1849. 10.1056/NEJMoa032922 15115830

[B24] Gong B. Legault D. Miki T. Seino S. Renaud J. M. (2003). KATP channels depress force by reducing action potential amplitude in mouse EDL and soleus muscle. Am. J. Physiol. 285, C1464–C1474. 10.1152/ajpcell.00278.2003 12917105

[B25] Gong B. Miki T. Seino S. Renaud J. M. (2000). A K(ATP) channel deficiency affects resting tension, not contractile force, during fatigue in skeletal muscle. Am. J. Physiol. Cell. Physiol. 279, C1351–C1358. 11029282 10.1152/ajpcell.2000.279.5.C1351

[B26] Gramolini A. Renaud J. M. (1997). Blocking ATP-sensitive K^+^ channel during metabolic inhibition impairs muscle contractility. Am. J. Physiol. 272, C1936–1946. 9227423 10.1152/ajpcell.1997.272.6.C1936

[B27] Greiff K. Mathiassen J. R. Misimi E. Hersleth M. Aursand I. G. (2015). Gradual reduction in sodium content in cooked ham, with corresponding change in sensorial properties measured by sensory evaluation and a multimodal Machine vision system. PLoS ONE 10:e0137805. 10.1371/journal.pone.0137805 26422367 PMC4589381

[B28] Harakalova M. van Harssel J. J. Terhal P. A. van Lieshout S. Duran K. Renkens I.. (2012). Dominant missense mutations in ABCC9 cause Cantu' syndrome. Nat. Genet. 44, 793–796. 10.1038/ng.2324 22610116

[B29] Hocquette J.-F. Bernard-Capel C. Vidal V. Jesson B. Levéziel H. Renand G.. (2012). The GENOTEND chip: a new tool to analyse gene expression in muscles of beef cattle for beef quality prediction. BMC Vet. Res. 8:135. 10.1186/1746-6148-8-135 22894653 PMC3438070

[B30] Inagaki N. Gonoi T. Clement J. P. Namba N. Inazawa J. Gonzales G.. (1995). Reconstitution of IKATP: an inward rectifier subunit plus the sulfonylurea receptor. Science 270, 1166–1170. 10.1126/science.270.5239.1166 7502040

[B31] Inagaki N. Gonoi T. Clement J. P. Wang C. Z. Aguilar-Bryan L. Bryan J.. (1996). A family of sulfonylurea receptors determines the pharmacological properties of ATP-sensitive K^+^ channels. Neuron 16, 1011–1017. 10.1016/S0896-6273(00)80124-5 8630239

[B32] Kane G. C. Behfar A. Yamada S. Perez-Terzic C. O'Cochlain F. Reyes S.. (2004). ATP-sensitive K^+^ channel knockout compromises the metabolic benefit of exercise training, resulting in cardiac deficits. Diabetes 53, 169–175. 10.2337/diabetes.53.suppl_3.S169 15561907

[B33] Kane G. C. Liu X. K. Yamada S. Olson T. M. Terzic A. (2005). Cardiac KATP channels in health and disease. J. Mol. Cell. Cardiol. 38, 937–943. 10.1016/j.yjmcc.2005.02.026 15910878 PMC2736958

[B34] Khanfar M. A. AbuKhader M. M. Alqtaishat S. Taha M. O. (2013). Pharmacophore modeling, homology modeling, and *in silico* screening reveal mammalian target of rapamycin inhibitory activities for sotalol, glyburide, metipranolol, sulfamethizole, glipizide, and pioglitazone. J. Mol. Graph. Model 42, 39–49. 10.1016/j.jmgm.2013.02.009 23545333

[B35] Kim M.-Y. Kim M. J. Yoon I. S. Ahn J. H. Lee S. H. Baik E. J.. (2006). Diazoxide acts more as a PKC-e activator, and indirectly activates the mitochondrial KATP channel conferring cardioprotection against hypoxic injury. Br. J. Pharmacol. 149, 1059–1070. 10.1038/sj.bjp.0706922 17043673 PMC2014640

[B36] Kwon G. Marshall C. A. Liu H. Pappan K. L. Remedi M. S. McDaniel M. L. (2006). Glucose-stimulated DNA synthesis through mammalian target of rapamycin (mTOR) is regulated by KATP channels effects on cell cycle progression in rodent islets. J. Biol. Chem. 281, 3261–3267. 10.1074/jbc.M508821200 16344552

[B37] Light P. E. Bladen C. Winkfein R. J. Walsh M. P. French R. J. (2000). Molecular basis of protein kinase C-induced activation of ATP-sensitive potassium channels. Proc. Natl. Acad. Sci. U.S.A. 97, 9058–9063. 10.1073/pnas.160068997 10908656 PMC16821

[B38] Liss B. Roeper J. (2001). Molecular physiology of neuronal K-ATP channels (review). Mol. Membr. Biol. 18, 117–127. 10.1080/09687680110047373 11463204

[B39] Martin T. P. Vailas A. C. Durivage J. B. Edgerton V. R. Castleman K. R. (1985). Quantitative histochemical determination of muscle enzymes: biochemical verification. J. Histochem. Cytochem. 33, 1053–1059. 10.1177/33.10.4045183 4045183

[B40] Mateescu R. G. Garmyn A. J. Tait R. G. Duan Q. Liu Q. Mayes M. S.. (2013). Genetic parameters for concentrations of minerals in longissimus muscle and their associations with palatability traits in angus cattle. J. Anim. Sci. 91, 1067–1075. 10.2527/jas.2012-5744 23230113

[B41] McTaggart J. S. Clark R. H. Ashcroft F. M. (2010). The role of the KATP channel in glucose homeostasis in health and disease: more than meets the islet. J. Physiol. 588(Pt 17), 3201–3209. 10.1113/jphysiol.2010.191767 20519313 PMC2976015

[B42] Megeney L. A. Neufer P. D. Dohm G. L. Tan M. H. Blewett C. A. Elder G. C.. (1993). Effects of muscle activity and fiber composition on glucose transport and GLUT-4. Am. J. Physiol. 264, E583–E593. 8476037 10.1152/ajpendo.1993.264.4.E583

[B43] Mele A. Buttiglione M. Cannone G. Vitiello F. Conte Camerino D. Tricarico D. (2012). Opening/blocking actions of pyruvate kinase antibodies on neuronal and muscular KATP channels. Pharmacol Res. 66, 401–408. 10.1016/j.phrs.2012.07.007 22967932

[B44] Mele A. Calzolaro S. Cannone G. Cetrone M. Conte D. Tricarico D. (2014b). Database search of spontaneous reports and pharmacological investigations on the sulfonylureas and glinides-induced atrophy in skeletal muscle. Pharmacol. Res. Perspect. 2, e00028. 10.1002/prp2.28 25505577 PMC4186404

[B45] Mele A. Camerino G. M. Calzolaro S. Cannone M. Conte D. Tricarico D. (2014a). Dual response of the KATP channels to staurosporine: a novel role of SUR2B, SUR1 and Kir6.2 subunits in the regulation of the atrophy in different skeletal muscle phenotypes. Biochem. Pharmacol. 91, 266–275. 10.1016/j.bcp.2014.06.023 24998494

[B46] Mohammed Abdul K. S. Jovanović S. Du Q. Sukhodub A. Jovanović A. (2015). Mild hypoxia *in vivo* regulates cardioprotective SUR2A: a role for Akt and LDH. Biochim. Biophys. Acta. 1852, 709–719. 10.1016/j.bbadis.2015.01.001 25576887 PMC4547089

[B47] Nelson P. T. Jicha G. A. Wang A.-X. Ighodaro E. Artiushin S. Nichols C. G.. (2015). ABCC9/SUR2 in the brain: implications for hippocampal sclerosis of aging and a potential therapeutic target. Ageing Res. Rev. 24, 111–125. 10.1016/j.arr.2015.07.007 26226329 PMC4661124

[B48] Nichols C. G. Singh G. K. Grange D. K. (2013). KATP channels and cardiovascular disease: suddenly a syndrome. Circ. Res. 112, 1059–1072. 10.1161/CIRCRESAHA.112.300514 23538276 PMC3660033

[B49] Nolin F. Michel J. Wortham L. Tchelidze P. Banchet V. Lalun N.. (2016). Stage-specific changes in the water, Na+, Cl- and K+ contents of organelles during apoptosis, demonstrated by a targeted cryo correlative analytical approach. PLoS ONE 11:e0148727. 10.1371/journal.pone.0148727 26866363 PMC4807926

[B50] Olson T. M. Terzic A. (2010). Human KATP channelopathies: diseases of metabolic homeostasis. Pflugers Arch. Eur. J. Physiol. 460, 295–306. 10.1007/s00424-009-0771-y 20033705 PMC2883927

[B51] Picard B. Duris M. Jurie C. (1998). Classification of bovine muscle fibres by different histochemical techniques. Histochem. J. 30, 473–479. 10.1023/A:1003207922944 10192530

[B52] Picard B. Gagaoua M. Micol D. Cassar-Malek I. Hocquette J. F. Terlouw C. E. (2014). Inverse relationships between biomarkers and beef tenderness according to contractile and metabolic properties of the muscle. J. Agric. Food Chem. 62, 9808–9818. 10.1021/jf501528s 25175407

[B53] Rexroad C. E. III Bennett G. L. Stone R. T. Keele J. W. Fahrenkrug S. C. Freking B. A.. (2001). Comparative mapping of BTA15 and SA11 including a region containing a QTL for meat tenderness. Mamm. Genome 12, 561–565. 10.1007/s0033500-20028 11420620

[B54] Ronda A. C. Buitrago C. Boland R. (2010). Role of estrogen receptors, PKC and Src in ERK2 and p38 MAPK signalling triggered by 17β-estradiol in skeletal muscle cells. J. Steroid Biochem. Mol. Biol. 122, 287–294. 10.1016/j.jsbmb.2010.05.002 20478382

[B55] Rottlaender D. Boengler K. Wolny M. Schwaiger A. Motloch L. J. Ovize M.. (2012). Glycogensynthase kinase 3β transfers cytoprotective signalling through connexin 43 onto mitochondrial ATP-sensitive K^+^ channels. Proc. Natl. Acad. Sci. U.S.A. 109, E242–E251. 10.1073/pnas.1107479109 22238425 PMC3277155

[B56] Schiaffino S. Gorza L. Sartore S. Saggin L. Ausoni S. Vianello M.. (1989). Three myosin heavy chain isoforms in type 2 skeletal muscle fibers. J. Muscle Res. Cell. Motil. 10, 197–205. 10.1007/BF01739810 2547831

[B57] Selivanov V. A. Alekseev A. E. Hodgson D. M. Dzeja P. P. Terzic A. (2004). Nucleotide-gated KATP channels integrated with creatine and adenylate kinases: amplification, tuning and sensing of energetic signals in the compartmentalized cellular environment. Mol. Cell. Biochem. 256–257, 243–256. 10.1023/B:MCBI.0000009872.35940.7d 14977185 PMC2760266

[B58] Stoller D. Pytel P. Katz S. Earley J. U. Collins K. Metcalfe J.. (2009). Impaired exercise tolerance and skeletal muscle myopathy in sulfonylurea receptor-2 mutant mice. Am. J. Physiol. 297, R1144–R1153. 10.1152/ajpregu.00081.2009 19675276 PMC2763822

[B59] Tateo A. De Palo P. Quaglia N. C. Centoducati P. (2007). Some qualitative and chromatic aspects of thawed buffalo (*Bubalus bubalis*) meat. Meat Sci. 76, 352–358. 10.1016/j.meatsci.2006.12.003 22064306

[B60] Thabet M. Miki T. Seino S. Renaud J. M. (2005). Treadmill running causes significant fiber damage in skeletal muscle of KATP channel-deficient mice. Physiol. Genomics 22, 204–212. 10.1152/physiolgenomics.00064.2005 15914579

[B61] Tizioto P. C. Gasparin G. Souza M. M. Mudadu M. A. Coutinho L. L. Mourão G. B.. (2013). Identification of KCNJ11 as a functional candidate gene for bovine meat tenderness. Physiol. Genomics 45, 1215–1221. 10.1152/physiolgenomics.00137.2012 24151244

[B62] Tizioto P. C. Gromboni C. F. de Araujo Nogueira A. R. de Souza M. M. de Alvarenga Mudadu M. Tholon P.. (2014). Calcium and potassium content in beef: influences on tenderness and associations with molecular markers in Nellore cattle. Meat Sci. 96, 436–440. 10.1016/j.meatsci.2013.08.001 23995697

[B63] Tricarico D. Camerino D. C. (1994). ATP-sensitive K^+^ channels of skeletal muscle fibers from young adult and aged rats: possible involvement of thiol-dependent redox mechanisms in the age-related modifications of their biophysical and pharmacological properties. Mol. Pharmacol. 46, 754–761. 7969056

[B64] Tricarico D. Mallamaci R. Barbieri M. Conte Camerino D. (1997a). Modulation of ATP-sensitive K^+^ channel by insulin in rat skeletal muscle fibers. Biochem. Biophys. Res. Commun. 232, 536–539. 10.1006/bbrc.1997.6320 9125217

[B65] Tricarico D. Mele A. Camerino G. M. Bottinelli R. Brocca L. Frigeri A.. (2010). The KATP channel is a molecular sensor of atrophy in skeletal muscle. J. Physiol. 588(Pt 5), 773–784. 10.1113/jphysiol.2009.185835 20064856 PMC2834937

[B66] Tricarico D. Mele A. Camerino G. M. Laghezza A. Carbonara G. Fracchiolla G.. (2008a). Molecular determinants for the activating/blocking actions of the 2H-1,4-benzoxazine derivatives, a class of potassium channel modulators targeting the skeletal muscle KATP channels. Mol. Pharmacol. 74, 50–58. 10.1124/mol.108.046615 18403717

[B67] Tricarico D. Mele A. Liss B. Ashcroft F. M. Lundquist A. L. Desai R. R.. (2008b). Reduced expression of Kir6.2/SUR2A subunits explains KATP deficiency in K+-depleted rats. Neuromuscul. Disord. 18, 74–80. 10.1016/j.nmd.2007.07.009 17825556

[B68] Tricarico D. Mele A. Lundquist A. L. Desai R. R. George A. L. Jr. Conte Camerino D. (2006). Hybrid assemblies of ATP-sensitive K^+^channels determine their muscle-type-dependent biophysical and pharmacological properties. Proc. Natl. Acad. Sci. U.S.A. 103, 1118–1123. 10.1073/pnas.0505974103 16418275 PMC1347972

[B69] Tricarico D. Montanari L. Conte Camerino D. (2003). Involvement of 3Na^+^/2K^+^ ATP-ase and Pi-3 kinase in the response of skeletal muscle ATP-sensitive K+ channels to insulin. Neuromuscul. Disord. 13, 712–719. 10.1016/S0960-8966(03)00095-6 14561494

[B70] Tricarico D. Petruzzi R. Conte Camerino D. (1997b). Different sulfonylurea and ATP sensitivity characterizes the juvenile and the adult form of KATP channel complex of rat skeletal muscle. Eur. J. Pharmacol. 321, 369–378. 10.1016/S0014-2999(96)00965-X 9085050

[B71] Tricarico D. Pierno S. Mallamaci R. Brigiani G. S. Capriulo R. Santoro G.. (1998). The biophysical and pharmacological characteristics of skeletal muscle ATP- sensitive K^+^ channels are modified in K^+^-depleted rat, an animal model of hypokalemic periodic paralysis. Mol. Pharmacol. 54, 197–206. 9658206 10.1124/mol.54.1.197

[B72] Tricarico D. Rolland J. F. Cannone G. Mele A. Cippone V. Laghezza A.. (2012). Structural nucleotide analogs are potent activators / inhibitors of pancreatic β cell KATP channels: an emerging mechanism supporting their use as antidiabetic drugs. J. Pharmacol. Exp. Ther. 340, 266–276. 10.1124/jpet.111.185835 22028392

[B73] Tricarico D. Servidei S. Tonali P. Jurkat- Rott K. Camerino D. C. (1999). Impairment of skeletal muscle adenosine triphosphate-sensitive K^+^ channels in patients with hypokalemic periodic paralysis. J. Clin. Invest. 103, 675–682. 10.1172/JCI4552 10074484 PMC408119

[B74] Tsiani E. Ramlal T. Leiter L. Klip A. Fantus I. (1995). Stimulation of glucose uptake and increased plasma membrane content of glucose transporters in L6 skeletal muscle cells by the sulfonylureas gliclazide and glyburide. Endocrinology 136, 2505–2512. 7750472 10.1210/endo.136.6.7750472

[B75] Vadlakonda L. Dash A. Pasupuleti M. Anil Kumar K. Reddanna P. (2013). The paradox of Akt-mTOR interactions. Front. Oncol. 165:1 10.3389/fonc.2013.00165 PMC3687210 23802099

[B76] Walsh H. Martins S. O'Neill E. E. Kerry J. P. Kenny T. Ward P. (2010). The effect of sodium lactate, potassium lactate, carrageenan, whey protein concentrate, yeast extract and fungal proteinases on the cook yield and tenderness of bovine chuck muscles. Meat Sci. 85, 230–234. 10.1016/j.meatsci.2010.01.003 20374890

[B77] Wasada T. (2002). Adenosine triphosphate-sensitive potassium (K(ATP)) channel activity is coupled with insulin resistance in obesity and type 2 diabetes mellitus. Intern. Med. 41, 84–90. 10.2169/internalmedicine.41.84 11868613

[B78] Wasada T. Yano T. Ohta M. Yui N. Iwamoto Y. (2001). ATP-Sensitive potassium channels modulate glucose transport in cultured human skeletal muscle cells. Endocr. J. 48, 369–375. 10.1507/endocrj.48.369 11523909

[B79] Wheeler A. Wang C. Yang K. Fang K. Davis K. Styer A. M.. (2008). Coassembly of different sulfonylurea receptor subtypes extends the phenotypic diversity of ATP-sensitive potassium (KATP) channels. Mol. Pharmacol. 74, 1333–1344. 10.1124/mol.108.048355 18723823 PMC2574914

[B80] Wojtovich A. P. Smith C. O. Haynes C. M. Nehrke K. W. Brookes P. S. (2013). Physiological consequences of complex II inhibition for aging, disease, and the mKATP channel. Biochim. Biophys. Acta 1827, 598–611. 10.1016/j.bbabio.2012.12.007 23291191 PMC3880126

[B81] Yancey J. W. S. Apple J. K. Meullenet J. F. Sawyer J. T. (2010). Consumer responses for tenderness and overall impression can be predicted by visible and near-infrared spectroscopy, Meullenet–Owens razor shear, and Warner–Bratzler shear force. Meat Sci. 85, 487–492. 10.1016/j.meatsci.2010.02.020 20416819

[B82] Zeng J. Wang G. Chen S. D. (2007). ATP-sensitive potassium channels: novel potential roles in Parkinson's disease. Neurosci. Bull. 23, 370–376. 10.1007/s12264-007-0055-5 18064068 PMC5550652

[B83] Zhang H. L. Bolton T. B. (1996). Two types of ATP-sensitive potassium channels in rat portal vein smooth muscle cells. Br. J. Pharmacol. 118, 105–114. 10.1111/j.1476-5381.1996.tb15372.x 8733582 PMC1909472

